# Evaluation of expression and function of the H^+^/*myo*-inositol transporter HMIT

**DOI:** 10.1186/1471-2121-10-54

**Published:** 2009-07-16

**Authors:** Elena Di Daniel, Man HS Mok, Emma Mead, Chiara Mutinelli, Erika Zambello, Laura L Caberlotto, Theresa J Pell, Christopher J Langmead, Ajit J Shah, Graham Duddy, James NC Kew, Peter R Maycox

**Affiliations:** 1 Psychiatry Discovery Technology Group, GlaxoSmithKline, New Frontiers Science Park, Harlow, UK; 2 Psychiatry Discovery Technology Group, GlaxoSmithKline, New Frontiers Science Park, Verona, Italy; 3 Core Discovery Technology Group, GlaxoSmithKline, New Frontiers Science Park, Harlow, UK; 4 Psychiatry Centre of Excellence for Drug Discovery, GlaxoSmithKline, New Frontiers Science Park, Harlow, UK

## Abstract

**Background:**

The phosphoinositide (PIns) signalling pathway regulates a series of neuronal processes, such as neurotransmitter release, that are thought to be altered in mood disorders. Furthermore, mood-stabilising drugs have been shown to inhibit key enzymes that regulate PIns production and alter neuronal growth cone morphology in an inositol-reversible manner. Here, we describe analyses of expression and function of the recently identified H^+^/*myo*-inositol transporter (HMIT) investigated as a potential regulator of PIns signalling.

**Results:**

We show that HMIT is primarily a neuronal transporter widely expressed in the rat and human brain, with particularly high levels in the hippocampus and cortex, as shown by immunohistochemistry. The transporter is localised at the Golgi apparatus in primary cultured neurones. No HMIT-mediated electrophysiological responses were detected in rat brain neurones or slices; in addition, inositol transport and homeostasis were unaffected in HMIT targeted null-mutant mice.

**Conclusion:**

Together, these data do not support a role for HMIT as a neuronal plasma membrane inositol transporter, as previously proposed. However, we observed that HMIT can transport inositol triphosphate, indicating unanticipated intracellular functions for this transporter that may be relevant to mood control.

## Background

Bipolar disorder is a severe psychiatric illness characterised by alternating episodes of mania and depression. Dysregulation of the PIns signalling pathway has been implicated in the pathophysiology of the disorder by magnetic resonance spectroscopy studies [1]. Furthermore, commonly prescribed drugs used to treat the illness (lithium, valproic acid and carbamazepine) alter neuronal growth cone morphology, a phenotype that is reversed by addition of extracellular *myo*-inositol [2,3].

Brain inositol levels are regulated by: a) transport from blood, b) recycling of intracellular inositol phosphates, and c) synthesis from glucose-6-phosphate to *myo*-inositol-1-phosphate (MIP) by the enzyme MIP-synthase [4,5]. MIP-synthase expression in the brain appears to be confined to the vasculature [5], suggesting that inositol synthesis may not play a key role in neuronal inositol signalling. Rather, an uptake system seems to be required to transport inositol across the plasma membrane into neurones, which may play a role in the regulation of signalling. Three *myo*-inositol transporters have been identified to date – the sodium *myo*-inositol transporters 1 and 2 (SMIT1 and SMIT2) [6] and the phylogenetically distant H^+^/*myo*-inositol transporter (HMIT) [7]. mRNA expression studies identified HMIT, but not SMIT1 or SMIT2, transcripts in rat neurones [3]. Also, HMIT expression appears highest in the hippocampus and cerebral cortex, areas which are implicated in mood disorders [8].

HMIT is a glycosylated protein containing three conserved internalisation signals: an endoplasmic reticulum (ER) retention signal in the N-terminal region, a dileucine internalisation signal and a tyrosine based internalisation motif at the C-terminal [7]. Mutation of the retention signals is required for plasma membrane localisation of the recombinant protein in *Xenopus *oocytes and mammalian cells, and this surface localisation correlated with functional inositol uptake into the cells [7]. Furthermore, HMIT is a symporter of *myo*-inositol and protons, since inositol uptake was only evident under acidic extracellular conditions and was associated with an inward electrical current and decreased intracellular pH [7]. It has been proposed that translocation of recombinant or endogenous HMIT to the plasma membrane may be activity-dependent following, for example, neuronal depolarisation or protein kinase C (PKC) activation [8].

Whilst HMIT represents an attractive candidate as a neuronal inositol transporter, it is unclear if HMIT contributes to the inositol-reversible effects of mood stabilisers on neurones [2] through transport of inositol into the cell. To address this question, we undertook studies to further characterise the localisation and functional properties of HMIT in recombinant systems and native tissue. We have analysed HMIT expression in rat and human brain tissue using immunohistochemistry and investigated the conditions necessary for its translocation to the plasma membrane in heterologous cells and cultured neurones. Functional expression was probed using [^3^H]-*myo*-inositol uptake, [^3^H]-cytidine diphosphate diacylglycerol (CDP-DAG) accumulation and whole-cell electrophysiology assays.

## Results

### Intracellular *myo*-inositol measurements in rat cortical neurones

**Figure 1:**
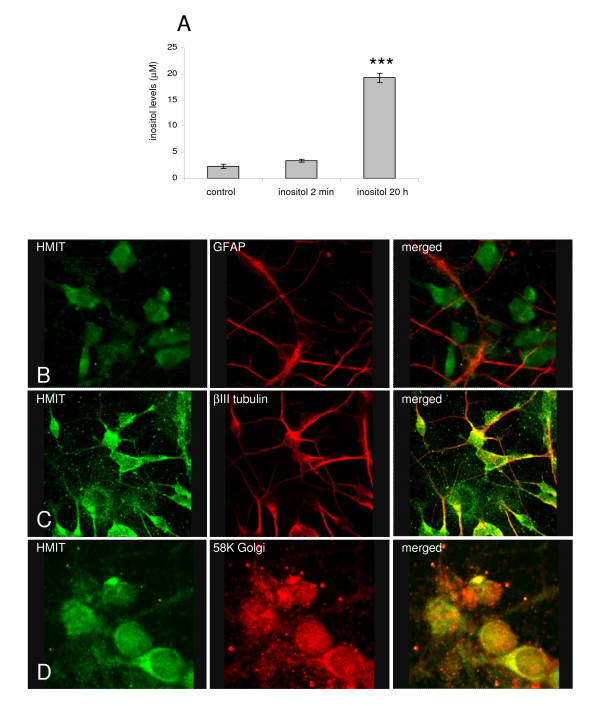
**Intracellular *myo*-inositol analysis and investigation of HMIT expression in rat dissociated neurones**. **A **Intracellular *myo*-inositol levels in rat cortical neurones incubated with *myo*-inositol showing increased intracellular inositol levels with time (n = 3 wells from one cell preparation). Primary cortical neurones were stained with anti-HMIT:21 (1:100) in combination with: **B **an anti-GFAP antibody (Abcam, 1:1000); **C **an anti-βIII tubulin antibody (Abcam, 1:1000); or **D **an anti-58K Golgi antibody (Abcam, 1:1000). Secondary antibodies Alexa Fluor anti-rabbit 488 and anti-mouse 633 were used. Neurones were imaged using a confocal microscope with a 63× water immersion objective.

To determine if extracellular *myo*-inositol can be taken up by neurones, we measured intracellular *myo*-inositol concentrations following incubation of cultured neurones with *myo*-inositol (1 mM) for 2 min or 20 h. After cell supernatant removal, cells were washed with ice-cold phosphate buffered saline (PBS) and lysed with ice-cold acetonitrile containing ammonium acetate; intracellular *myo*-inositol concentration was measured using liquid chromatography tandem mass spectrometry (LC-MS/MS). Intracellular inositol levels were found to be significantly increased after overnight extracellular application (19.25 ± 0.84 vs 2.33 ± 0.38 μM, mean ± sem; ***p < 0.001; Figure 1A), indicating that inositol can be taken up across the plasma membrane. Intracellular inositol concentration was not changed after 2 min incubation (3.43 ± 0.26 vs 2.33 ± 0.38 μM, mean ± sem; p = 0.07, one-way ANOVA followed by Dunnett's post-hoc test; Figure 1A). We subsequently proceeded to investigate whether HMIT is involved in the transport of *myo*-inositol into neurones by studying its expression and function.

### HMIT expression in the brain

In order to analyse HMIT localisation, we generated a specific antibody, as demonstrated by Western blotting, immunoprecipitation and immunocytochemistry (see Additional files 1 and 2). The cellular specificity and localisation of HMIT was analysed by confocal microscopy of rat primary cortical cultures co-stained with anti-HMIT:21 antibody and either a neuronal, astrocytic or Golgi marker. We observed no co-localisation of HMIT and the astrocytic marker GFAP (Figure 1B), indicating little or no expression of HMIT in primary astrocytes, which are a cell contaminant in the neuronal preparation. The images in Figure 1C show that HMIT co-localised with βIII tubulin in the cell body and neurites, indicating neuronal expression. Furthermore, the HMIT antibody co-localised with the Golgi marker 58K Golgi protein (Figure 1D), suggesting that HMIT is present in an intracellular compartment associated with the Golgi apparatus.

**Figure 2:**
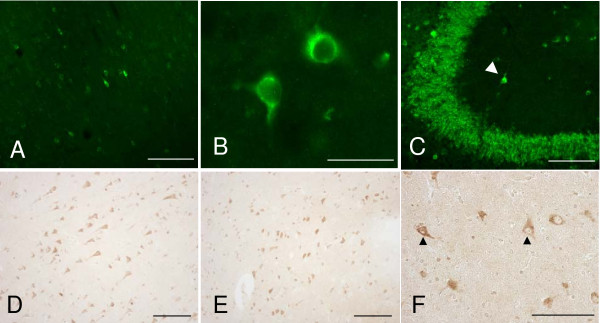
**Analysis of HMIT expression in rat and human brain**. Fluorescence microphotographs of adult male rat coronal sections immunolabelled with anti-HMIT antibody. HMIT-LI distribution in the secondary motor cortex (M2), at approximately +1.20 mm from Bregma at low (**A**) and high magnification (**B**); and in the CA3 hippocampal region, approximately -2.80 mm from Bregma (**C**); the arrowhead indicates a HMIT-positive interneuron; Scale bar = 100 μm (**A, C**) and 50 μm (**B**). Microphotographs of formalin-fixed paraffin embedded sections of human brain areas immunolabelled with anti-HMIT antibody. HMIT staining is visible in cell bodies and projections of different regions of the human brain: cerebral cortex (**D**); hippocampus (**E**) and thalamus (**F**), the latter at higher magnification, in which two examples of HMIT positive staining are indicated by the arrowheads. Scale bar = 50 μm (**D, E, F**).

To investigate HMIT localisation in the rat brain, immunohistochemistry was performed in sagittal slices from adult rat brain with staining visualised using the Odyssey infra-red detection system (Li-Cor) (see Additional file 1). Staining was observed in the hippocampal region, with high expression levels of the HMIT protein in CA2-3 and dentate gyrus of the hippocampus (see Additional file 3). In addition, the immunising peptide effectively competed with the observed signal in rat brain (data not shown). Subsequently, fluorescence and colorimetric immunohistochemistry were performed to investigate the detailed anatomical and cellular distribution of HMIT-like immunoreactivity (HMIT-LI) in rat and human brain. HMIT-LI appeared to be distributed in discrete regions of the rat brain with a strong expression in the hippocampus, particularly in the CA2-3 regions and in the dentate gyrus (Figure 2C). Moderate HMIT expression was observed in the cerebral motor cortex (Figure 2A and 2B). In the human brain, a moderate to high HMIT-LI signal was detected in the cerebral cortex (Figure 2D) and hippocampus (Figure 2E). As shown in Figure 2B (rat) and in Figure 2F (human) the HMIT-LI seems to be localised predominantly intracellularly.

### Analysis of HMIT triple mutant

**Figure 3:**
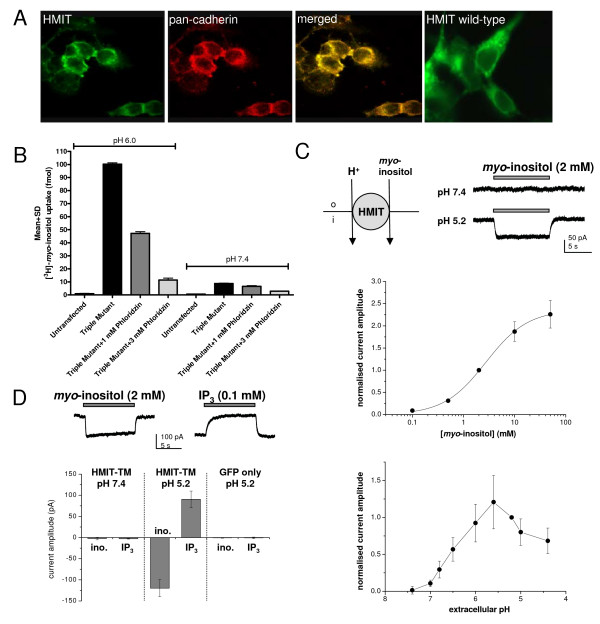
**HMIT activity in HEK293T cells transiently expressing HMIT triple mutant at the plasma membrane**. **A **HMIT triple mutant is expressed at the plasma membrane in HEK cells as indicated by co-localisation with pan-cadherin antibody (Abcam, 1:1000) observed by confocal microscopy, in contrast to wild-type HMIT, which is mainly localised intracellularly. **B **[^3^H]-*myo*-inositol uptake and its concentration dependent inhibition using the non-selective HMIT inhibitor phloridzin were demonstrated at pH 6.0. **C **Top left: schematic diagram illustrating the proposed electrogenic transport by HMIT. Top right: representative traces of inositol-evoked currents recorded in a HEK293T cell transiently transfected with HMIT triple mutant (HMIT-TM). Middle: concentration-response relationship for *myo*-inositol in the HMIT triple mutant at pH 5.2 (n = 5–9 cells per data point). Bottom: pH sensitivity of currents evoked by *myo*-inositol (2 mM), recordings were made in HEK293T cells transfected with the HMIT triple mutant (n = 4–7 per data point). **D **The HMIT triple mutant was also demonstrated to transport IP_3_. Similar to observations with *myo*-inositol, the electrogenic transport of IP_3 _was observed at pH 5.2 and not at pH 7.4 (n = 4–6 per data point).

We first studied and characterised HMIT function using the HMIT triple mutant (generated as described by [7]). It was previously shown that recombinant HMIT is localised in the cytoplasm of HEK293 cells and that expression of an HMIT triple mutant was required to achieve plasma membrane localisation [7]. In order to confirm and extend these findings, we transfected HEK293 cells with pcDNA3.1/V5-His-TOPO-HMIT triple mutant and analysed HMIT localisation by immunocytochemistry using the anti-HMIT:21 antibody and an anti-pan cadherin antibody to confirm plasma membrane localisation (Figure 3A). HMIT triple mutant co-localised with pan-cadherin staining, indicating that the HMIT triple mutant is expressed at the plasma membrane, in contrast to the wild-type recombinant HMIT. In the [^3^H]-inositol uptake assay using HEK293T cells transfected with the HMIT triple mutant, inositol uptake activity was observed at pH 6.0 and this was partially reversed by treatment with the non-selective inhibitor phloridzin (53 ± 1.4% inhibition with 1 mM phloridzin, 89 ± 1.5% inhibition with 3 mM phloridzin; n = 2 independent experiments). In contrast, the activity measured at pH 7.4 was much lower (100 ± 1.1 fmol at pH 6.0 versus 8.8 ± 0.26 fmol at pH 7.4; n = 2 independent experiments) (Figure 3B). These data indicate that the HMIT triple mutant is expressed at the plasma membrane and is functional at pH 6.0.

HMIT activity was further analysed using patch-clamp electrophysiology. Whole-cell voltage-clamp recordings were made from EGFP-positive HEK293 cells transiently co-transfected with the HMIT triple mutant and EGFP plasmids. *Myo*-inositol (2 mM) was applied by fast perfusion for 10 s at 60 s intervals under various conditions. At the holding potential of -60 mV, no current responses were observed at extracellular pH 7.4 (Figure 3C). However, when the extracellular pH was 5.2, *myo*-inositol evoked an inward, non-desensitising current. This response was independent of extracellular Na^+ ^and showed weak voltage-dependence (data not shown). The magnitude of the inositol-evoked currents increased as the extracellular environment was further acidified and reached a plateau level between pH 6 and pH 5 (Figure 3C). Characterising the concentration-response relationship of *myo*-inositol gave an EC_50 _of 2.7 mM, with a Hill slope of 1.0 (n = 5–9 per data point; Figure 3C). This contrasts with the affinity constant (Km) of 100 μM as reported by Uldry *et al. *(2001) using oocytes. In this study, the inositol-evoked currents were sensitive to inhibition by phloridzin (1 mM; 64.0 ± 3.1% inhibition, n = 4) and a similar level of inhibition was also observed in the absence of extracellular Na^+ ^(48.0 ± 6.7% inhibition, n = 4; data not shown). In addition we investigated whether inositol triphosphate (IP_3_) is also a substrate for HMIT. In HEK293T cells transiently transfected with the HMIT triple mutant, application of IP_3 _(0.1 mM) did not generate any responses at pH 7.4. When the extracellular pH was set at 5.2, application of IP_3 _evoked an outward current (+91 ± 20 pA, n = 4) while *myo*-inositol (2 mM) induced a current of opposite polarity (-120 ± 20 pA, n = 8; Figure 3D). The opposite direction of the net current can be explained by the negatively charged IP_3 _compared with the neutral inositol molecule. We confirmed that these responses were mediated by the HMIT triple mutant as no currents were observed at pH 5.2 when the experiment was repeated using HEK293T cells transfected with EGFP only as a control (IP_3_, -0.7 ± 1.8 pA, n = 5; *my*o-inositol, -0.1 ± 0.9 pA, n = 5).

### HMIT-mediated currents in rat neurones

**Figure 4:**
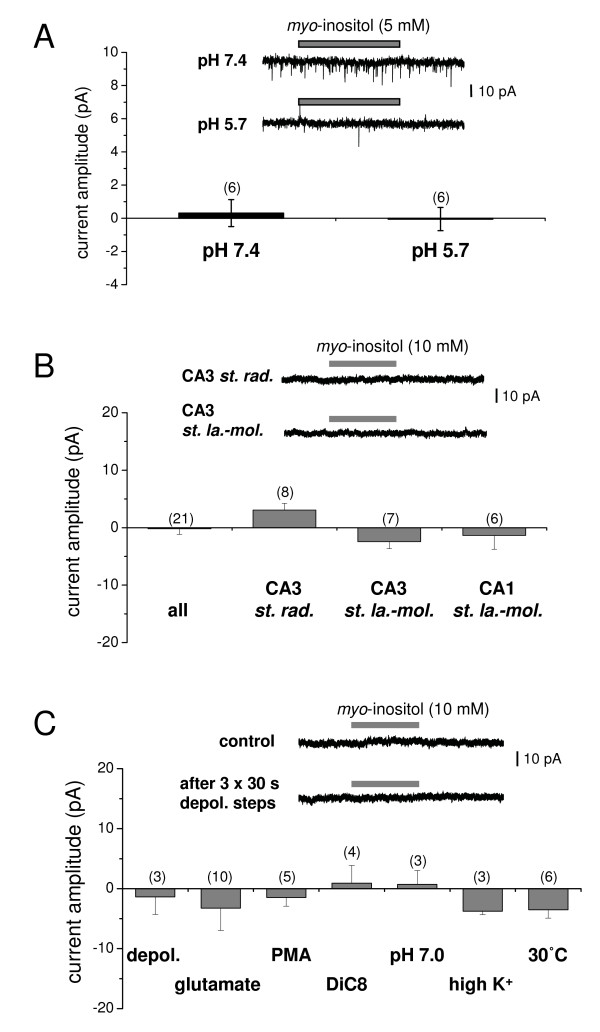
**Absence of functional native plasma membrane HMIT with electrophysiological analysis**. **A **Summary data showing that *myo*-inositol (5 mM) did not evoke any currents at either pH 5.7 or 7.4 in rat cultured cortical neurones. *Inset*, representative traces of typical recordings. The downward deflections are spontaneously-occurring synaptic currents because TTX was not included as the effects on HMIT-mediated responses were unknown (n numbers in parentheses). **B **Summary data showing that micro-pressure application of *myo*-inositol (10 mM) did not induce any current responses in interneurones in different regions of rat hippocampal slices. *Inset*, representative traces of typical recordings from a CA3 *stratum radiatum *and a CA3 *stratum lacunosum-moleculare *interneurone. **C **Summary data showing that inositol-evoked currents were not observed following a variety of stimulation protocols. (**Depol.**: three 30 s steps of depolarising current injection in current-clamp mode to induce action potential firing, **glutamate**, **PMA**, **DiC8**, **pH 7.0**: pressure application of *myo*-inositol dissolved in pH 7.0 extracellular solution; **high K^+^**, **30°C**: pre-incubation of slices at 30°C for >2 h before recording). *Inset*, representative traces of a typical recording of a CA3 *stratum lacunosum-moleculare *interneurone before (control) and after three depolarising steps. The stimulation paradigm did not promote responses to *myo*-inositol application.

Following characterisation of the HMIT triple mutant, we investigated rat brain tissue for endogenous HMIT-mediated responses. Whole-cell recordings (holding potential -60 mV) were carried out in cultured cortical neurones and to ensure HMIT was not unknowingly inhibited, we did not include tetrodotoxin (TTX) to block spontaneously occurring synaptic currents. At both extracellular pH 7.4 and 5.7, applications of *myo*-inositol (5 mM) did not evoke any current responses (0.3 ± 0.8 pA, -0.1 ± 0.7 pA, respectively; n = 6; Figure 4A).

Our localisation data showed HMIT staining in interneurones of the CA1 and CA3 regions of the hippocampus (Figure 2C arrowhead). To test for HMIT function in these cells, we made whole-cell voltage-clamp recordings in acute rat hippocampal slices and applied *myo*-inositol (10 mM) onto the cell bodies by micro-pressure application. As illustrated in Figure 4B, we found no inositol-evoked currents in interneurones located in the CA3 *stratum radiatum*, CA3 *stratum lacunosum-moleculare *or CA1 *stratum lacunosum-moleculare*. To test if neuronal activity is required for HMIT translocation to the plasma membrane, we stimulated the slices using various conditions and found no current responses on application of *myo*-inositol (Figure 4C). These data obtained in rat dissociated neurones as well as in rat slices suggest that functional HMIT is not present at the plasma membrane of the soma.

### Inositol studies in HMIT null-mutant neurones

**Figure 5:**
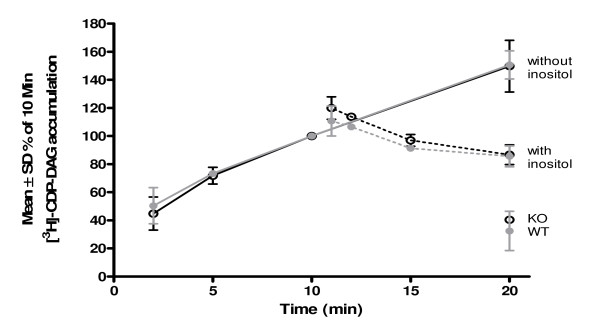
**[^3^H]-CDP-DAG accumulation in HMIT null-mutant (KO) and wild-type (WT) mouse cortical neurones**. [^3^H]-CDP-DAG accumulation increased with time in WT (black lines) and HMIT KO (grey lines) neurones stimulated with 1 mM CCh in the presence of 10 mM LiCl at pH 7.4. There was no difference in the reversal of [^3^H]-CDP-DAG accumulation in WT (black dashed lines) and HMIT KO (grey dashed lines) neurones following application of 3 mM extracellular *myo*-inositol.

We further investigated the functional expression of HMIT in cultured neurones using the [^3^H]-CDP-DAG accumulation assay by comparing wild-type (WT) and HMIT null-mutant (KO) mice. Cells were stimulated with the muscarinic receptor agonist carbachol (CCh) to activate PIns signalling, and treated with LiCl to block the enzyme inositol monophosphatase, therefore reducing intracellular inositol with the consequent accumulation of CDP-DAG. [^3^H]-CDP-DAG accumulation was measured in cortical neurones from WT and KO mice (Figure 5). Application of extracellular *myo*-inositol clearly reversed [^3^H]-CDP-DAG accumulation, at physiological pH in WT neurones. No difference was observed between WT and HMIT null-mutant neurones, thus indicating that inositol enters the cell despite genetic ablation of HMIT (see Additional files 1 and 4) [9].

## Discussion

In this study we show that HMIT is a neurone-specific protein that is widely expressed in the brain. The brain distribution is supported by the data from the Allen Mouse Brain Atlas  and Northern blot analysis [7]. The cellular localisation data obtained in the present study, however, contrast with that reported by Uldry *et al. *[7] which suggested widespread astrocytic localisation. The latter is inconsistent with our immunohistochemical data and also the QPCR data of Cahoy *et al. *[10], which suggest a neuronal expression (up to mouse postnatal day 30). The discrepancy may be explained by the relative specificity of the antibodies used in the two studies. We confirmed antibody specificity for HMIT using a series of methods including tagged-HMIT and peptide competition assays. Intracellular localisation was investigated both *in vitro *in primary neurones and *ex vivo *in rat brain sections. We showed that HMIT is co-localised with a Golgi marker and is not present at the cell surface or in neuronal processes. The Golgi localisation data support findings by [7,8], who observed intracellular staining as demonstrated by co-localisation with the intracellular ER marker calreticulin. We also demonstrated HMIT expression in several regions of the human brain by immunohistochemistry, with the signal being consistent with an intracellular localisation.

In agreement with previous studies, triple mutant HMIT is localised at the plasma membrane when overexpressed in HEK293 cells. The triple mutant was functional, as shown by both electrophysiology and inositol uptake assays, and we confirmed the characteristics of HMIT inositol transport – pH dependence, sodium independence, phlorizidin sensitivity – first reported by Uldry *et al. *[7]. We extended our analysis to rat primary neurones and hippocampal tissue slices where we were unable, even after stimulation, to demonstrate inositol-induced currents. We recognised that as pressure application of inositol was aimed at the soma we cannot exclude that translocation of HMIT may occur further away in the processes. However, in our localisation studies there was clear staining in the soma with no indication that HMIT is enriched in the processes. We used acutely prepared brain slices and primary dissociated neurones in contrast to Uldry *et al. *(2004) who showed translocation in rat brain aggregates, and preferential expression in regions of nerve growth and varicosities in primary neurones overexpressing recombinant HMIT. The lack of stimulation-induced HMIT activity was unlikely to be due to insufficient time course as translocation of HMIT was observed after 10 min application of PMA by [8], while we did not observe inositol-induced currents even after 30–60 min stimulation of slices. Although experiments were performed at room temperature, we did not observe any difference when slices were incubated at 30°C for more than 2 h suggesting that function was not affected by lower temperature.

Our data on localisation and function suggest that HMIT is not a plasma membrane transporter. The novel observation that HMIT can also transport IP_3 _provides an interesting alternative function for this protein. We speculate that HMIT may play a role in intracellular regulation of inositol and phosphoinositides, e.g. by sequestering IP_3 _into vesicles, possibly affecting intracellular calcium signalling.

## Conclusion

Overall, our study has shown that HMIT is expressed at the plasma membrane and is able to transport inositol (at pH 6.0 or below) but only under artificial conditions whereby it lacks intracellular retention motifs. In contrast, the native protein has secondary structural features consistent with intracellular localisation and retention and immunocytochemical analysis confirms its localisation in the ER/Golgi network. We could not demonstrate any activity-dependent inositol transport *in vitro *or *ex vivo *in neurones. These data suggest that HMIT is not involved in the neuronal transport of inositol from the extracellular environment and the mechanism underlying this process remains to be identified.

## Methods

All chemicals, unless otherwise stated, were purchased from Sigma.

All animal experimental procedures were performed according to either the UK Animals (Scientific Procedures) Act of 1986 or according to the Italian law (art. 7, Legislative Decree No. 116, 27 January 1992), which acknowledged the European Directive 86/609/EEC, and GlaxoSmithKline policy on the care and use of laboratory animals and related codes of practice. All efforts were made to minimise the number of animals used.

All human material used in this research was collected and used in compliance with the Declaration of Helsinki and in accordance with any relevant laws. In all cases informed consent has been obtained from the donor or donor's next of kin.

HMIT nucleotide sequence [NH_001033633] was used to generate HMIT null-mutant mice.

### Plasmids

The plasmids used were pcDNA3.1/V5-His-TOPO (Invitrogen) containing rat wild-type or rat HMIT triple mutant DNAs, all generated at GlaxoSmithKline. HMIT was cloned in frame with the V5-His tags by deleting the stop codon. The triple mutant, as described previously [7], was generated by site-directed mutagenesis using the QuikChange^® ^Multi Site-Directed Mutagenesis Kit (Stratagene) following manufacturer's instructions.

### HMIT antibody generation

Rabbit polyclonal affinity purified antibody anti-HMIT #21 (anti-HMIT:21) was produced by Open Biosystems. The following conserved peptide (human, rat and mouse) coupled to KLH was chosen: RWLIQKGQTQKARRILS [rat accession number AJ315643], which is located in the intracellular loop between transmembrane domains 6 and 7.

### Cell culture and transient transfection

Human embryonic kidney (HEK) 293 cells were cultured in MEM (Invitrogen), 10% (v/v) foetal bovine serum (FBS) (Invitrogen), 1% (v/v) MEM non-essential amino-acids (Invitrogen), 2% (v/v) L-glutamine (Invitrogen).

Cells were seeded at 30,000–50,000 cells per well (in 24-well plate format) or at 200,000 cells per well (in 6-well plate format) and grown for 24 h before transfection using Fugene 6 (Roche) according to manufacturer's instructions. Cells were fixed or lysed 48 h post-transfection.

### Rat and mouse cortical neurone preparation

Cortical neurones were cultured from gestational day 18 rat foetal tissue of Sprague Dawley rats [9] or gestation day 15 mouse foetal tissue. Briefly, cerebral cortices were collected in Hank's balanced salts solution, the meninges removed, and cortex dissected and dissociated with a papain tissue dissociation kit (Worthington) following the manufacturer's instructions. Cells were resuspended in Neurobasal medium (it contains 40 μM inositol) containing 2% (v/v) B27 supplement, 1 mM sodium pyruvate, 2 mM L-glutamine, 100 U/ml penicillin and 50 μg/ml streptomycin (media components from Invitrogen).

### Rat hippocampal slice preparation

Sprague-Dawley rats (postnatal day (P) 16–27) were deeply anaesthetised with isoflurane by inhalation and the brains were removed following decapitation. Using a vibratome (HM650V, Carl Zeiss Ltd.), horizontal hippocampal slices (250–300 μm thick) were cut in ice-cold artificial cerebrospinal fluid (aCSF) of the following composition: 125 mM NaCl, 2.5 mM KCl, 26 mM NaHCO_3_, 1.25 mM NaH_2_PO_4_.H_2_O, 25 mM glucose, 1 mM CaCl_2_, 2 mM MgCl_2_; bubbled with 95% O_2_/5% CO_2_. Slices were incubated at room temperature in aCSF for an hour before experimentation and used for up to 8 hours after preparation.

### Intracellular *myo*-inositol measurement (Table 1)

800,000 cortical neurones/well were plated in 6-well plates. After 8–9 days in culture the neurones were stimulated with inositol, lysed in 500 μl of ice-cold acetonitrile with 100 mM ammonium acetate (90:10, v/v), briefly spun down and supernatant analysed. A standard curve was generated using pure *myo*-inositol (10–0.1 μM). A Jasco binary gradient HPLC system was used. Eluates were detected using an Applied Biosystems Sciex API-4000 triple-quadrupole mass spectrometer equipped with a TurboIonSpray ion-source. The operating parameters of the ion-source, including analyte-dependent and source-dependent were optimised to obtain the optimum performance from the mass spectrometer for the analysis of *myo*-inositol. The sensitivity of detection for *myo*-inositol in the negative ion mode was found to be higher than in positive mode. The collision associated dissociation of *myo*-inositol precursor at *m/z *(mass/charge) 179 produced abundant ions at *m/z *161, 117, 99 and 87. Of these product ions *m/z *179→87 transition was selected as it provided the greatest selectivity. For [^2^H_6_]-*myo*-inositol (CDN isotopes) the best signal for precursor-to-product transition was *m/z *185→167. The optimum values for declustering potential, collision energy, entrance potential and collision exit potential for the precursor-to-product ion transitions selected are listed below. The source dependent parameters for *myo*-inositol consisted of collision gas, curtain gas, ion spray gas 1 and 2, ionspray voltage and the temperature of the heater gas, with optimum values of 6, 10, 20, 30, -4.5 kV and 600°C, respectively. An aliquot (5 μl) of *myo*-inositol was loaded onto a 150 × 2.1 mm i.d. Luna HILIC column (Phenomenex). The column temperature was maintained at 35°C. A linear gradient elution profile was used for the separation of *myo*-inositol. The mobile-phase consisted of eluent 'A', composed of a mixture of acetonitrile and 100 mM ammonium acetate buffer (90:10%, (v/v)), and eluent 'B', composed of acetonitrile, water and 100 mM ammonium acetate buffer (50:40:10%, (v/v)). A flow rate of 0.43 ml/min was used. The following elution profile was used: 0.0 min – 100% A; 2.5 min – 100% A; 12.0 min – 50% A and 50% B (linear gradient from 2.5 to 12.0 min); 12.9 min – 50% A and 50% B; 13.0 min – return to 100% A; hold for 7 min before proceeding to the next injection.

**Table 1:** Intracellular myo-inositol measurement

Analyte	Collision energy (eV)	Collision exit potential (V)	Declustering potential (V)	Entrance potential (V)
*myo*-inositol	-10.0	-5.0	-70.0	-10.0
[^2^H_6_]-*myo*-inositol	-18.0	-9.0	-70.0	-10.0

### Immunocytochemistry – primary neurones and cell lines

Cells were seeded at 30,000–50,000 cells per well in 24-well plate format on glass coverslips coated with poly-D-lysine. Following transfection, cells were fixed with 4% (w/v) paraformaldehyde (PFA) for 15 min at room temperature (RT). Cells were washed three times with PBS and permeabilised with 0.3% (v/v) Triton X100 for 5 min. 5% (v/v) normal goat serum (Chemicon) in PBS was used as blocking agent for 1 h. Primary antibodies were diluted in blocking solution and cells incubated overnight at 4°C with gentle shaking, followed by three washes with PBS. The secondary antibodies used were Alexa Fluor conjugated antibodies (Molecular Probes; 1:400), applied for 1 h at RT (in darkness). The coverslips were mounted onto glass slides with ProLong Gold antifade reagent with or without DAPI (Molecular Probes) to detect the cell nuclei. Cells were visualised using an Olympus BX51 microscope equipped with epifluorescent optics using Image-Pro Plus (Media Cybernetics) or Special Cell F (Olympus) software to analyse the images. For confocal imaging a Leica TLS SP confocal microscope with 63× and 100× water objectives was used with Leica confocal software.

### Immunohistochemistry – rat and human brain

The analysis of the HMIT protein distribution in rat brain was performed on adult animals anaesthetised with chloral hydrate (400 mg/kg) and transcardially perfused with saline for 5 min, followed by 15 min of fixation with 4% (w/v) PFA in PBS. Thirty μm-thick coronal brain sections were cut using a vibratome (Leica) and left free-floating in PBS. The slices were firstly exposed to antigen-retrieval, consisting of 30 min incubation at 80°C in 0.1 M Tris-HCl added with 1 mM EDTA, pH 9 and then blocked for 1 h in PBS supplemented with 3% (v/v) goat serum (Vector Laboratories), 0.3% (v/v) Triton X-100 (Sigma) and 0.02% (v/v) bovine serum albumin (BSA) at RT. The slices were incubated overnight at 4°C in the same buffer with anti-HMIT:21 at a dilution of 1:200 and then incubated in PBS with the fluorescent secondary antibody 488 goat anti-rabbit (Alexa-Fluor; Molecular Probes). Finally, slices were mounted on slides with Vectashield-mounting medium for fluorescence (Vector Laboratories), covered with a coverslip and visualised by fluorescent microscopy.

The distribution of the HMIT protein in the human brain was investigated in formalin-fixed and paraffin embedded (FFPE) tissues obtained from Zoion, Inc. as an anatomical gift, following approved ethical guidelines. Five μm-thick tissue slices were cut using a microtome (Micron) and collected on slides. The tissue sections were then processed through a de-paraffination consisting of a series of washes in xylene, mixtures of xylene and ethanol, and decreasing ethanol concentrations (100%, 95%, 70%, 50%). The sections were then exposed to heat-induced antigen retrieval in a microwave oven (2 cycles of 5 min with an output of 700 W with a 1 min interval between the two boiling cycles) in 0.1 M Tris-HCl with 1 mM EDTA, pH 9. The slices were then incubated for 10 min in 1% (v/v) H_2_O_2 _in PBS containing 0.05% (v/v) Tween 20 and then blocked for 1 h at RT in the same blocking buffer used for rat tissues. In the same buffer, the slices were incubated overnight at 4°C with anti-HMIT:21 antibody at a dilution of 1:200. Subsequently, tissue sections were incubated with biotinylated anti-rabbit IgG (H + L) affinity purified made in goat secondary antibody (Vector Laboratories) in PBS with 1% (v/v) goat serum and then exposed for 45 min to avidin-biotinylated peroxidase complex (ABC Standard, Vector Laboratories), before incubation in 3,3'-diaminobenzidine (DAB, Vector Laboratories) for 3 min followed by a series of dehydrating washes (increasing ethanol concentrations and xylene).

### [^3^H]-*myo*-inositol uptake in HEK293T cells

The method utilised was based on Uldry *et al. *[7]. HEK293T cells were seeded at 40,000 cells per well in 24-well poly-D-lysine-coated plate format. 24 h after seeding, cells were transiently transfected with HMIT triple mutant DNA using Fugene 6 (Roche) according to the manufacturer's instructions. 48 h after transfection, [^3^H]-*myo*-inositol uptake was measured at pH 6.0 in a total assay volume of 500 μl. Cells were washed twice with K5 buffer (5 mM KCl, 127 mM NMDG, 10 mM D-glucose, 1 mM MgCl_2_, 20 mM HEPES, 2.7 mM CaCl_2_; pH 7.4). Cells were pre-incubated for 10 min at 37°C with 0, 1 or 3 mM phloridzin, a non-selective HMIT inhibitor, in 450 μl of K5 buffer (5 mM KCl, 127 mM NMDG, 10 mM D-glucose, 1 mM MgCl_2_, 20 mM HEPES, 2.7 mM CaCl_2_; pH 6.0). Phloridzin was prepared as a 100× stock in neat ethanol, subsequently diluted in the assay buffer to give a final concentration of 1% ethanol. The reaction was started by the addition of 50 μl (5 μCi) of [^3^H]-*myo*-inositol (GE Healthcare)/2 mM cold *myo*-inositol mix, prepared in pH 6.0 K5 buffer. After 2 min the reaction was stopped by aspiration of the [^3^H]-*myo*-inositol and washing five times with 0.75 ml of ice-cold PBS. Cells were solubilised in 0.5 ml of 5% (w/v) sodium dodecyl sulfate (SDS) at 37°C for 30 min. Lysates were mixed with 4.5 ml of Ultima Gold XR (Perkin Elmer) scintillation liquid and radioactivity counted by scintillation spectroscopy (Tri-Carb 2800TR, Perkin Elmer). All data are reported as mean ± S.D.

### [^3^H]-CDP-DAG accumulation in mouse HMIT null-mutant cortical neurones

Mouse HMIT null-mutant and wild-type cortical neurones, prepared as described above, were seeded at 150,000 cells per well in 24-well poly-D-lysine-coated plates. Seven or nine days after plating, [^3^H]-CDP-DAG accumulation was measured using a method adapted from Atack *et al. *[11]. Media was removed and neurones pre-incubated with 300 μl/well of modified Krebs-Hensleit buffer (118 mM NaCl, 25 mM NaHCO_3_, 4.7 mM KCl, 1.3 mM CaCl_2_, 1.2 mM MgSO_4_, 1.2 mM KH_2_PO_4_, 5 mM HEPES, 10 mM glucose; pH 7.4) containing 0.3 μCi [^3^H]-5-cytidine (Sigma) for 1 h at 37°C. Endogenous muscarinic G-protein coupled receptors were stimulated with 1 mM CCh in the presence of the inositol monophosphatase and inositol polyphosphate 1-phosphatase inhibitor LiCl (10 mM), to prevent formation of *myo*-inositol, thus, resulting in [^3^H]-CDP-DAG accumulation. Experiments were performed over a time course in the absence or presence of 3 mM extracellular *myo*-inositol. Incubations were terminated by the addition of 1 ml of a mixture of methanol and chloroform (2:1, (v/v)) containing 1 M HCl to each well, which was left to extract for 5 min at RT. Well contents were transferred to centrifuge tubes; 310 μl of chloroform and 560 μl of 0.1 M HCl were added to aid phase separation. Samples were centrifuged at 0.1 rcf for 10 min at RT. An aliquot (400 μl) of the lower organic phase was removed, without disturbing the aqueous upper layer, transferred to scintillation vials and solvent was evaporated overnight. Ultima Gold XR scintillant liquid (4.5 ml) was then added and the samples counted by scintillation spectroscopy. All data are reported as mean ± S.D.

### Whole-cell patch-clamp recordings

HEK293 cells and cultured rat neurones, prepared as above, were perfused with an external solution containing: 145 mM NaCl, 2.5 mM KCl, 10 mM HEPES, 10 mM glucose, 2 mM CaCl_2_, 1 mM MgCl_2_, pH 7.4 with NaOH. In some experiments, NaCl was replaced with NMDG-Cl. Patch pipettes (5–10 MΩ tip resistance) were filled with: 135 mM KMeSO_4_, 10 mM HEPES, 4 mM NaCl, 4 mM Mg-ATP, 0.2 mM Na_2_GTP, 0.5 mM EGTA; pH 7.3; 280–300 mOsmol/kg. Membrane currents were recorded by whole-cell voltage-clamp using an Axopatch 200B amplifier and pClamp9 software (Molecular Devices). The holding membrane potential was set at -60 or -70 mV unless specified otherwise. Solutions containing test compounds were applied via a dual-barrel fast perfusion system (RSC-160; Biologic) and all experiments were conducted at RT.

Hippocampal slices were superfused (2–3 ml/min) at RT with aCSF: 125 mM NaCl, 2.5 mM KCl, 26 mM NaHCO_3_, 1.25 mM NaH_2_PO_4_.H_2_O, 25 mM glucose, 2 mM CaCl_2_, 1 mM MgCl_2_; pH 7.3 when bubbled with 95% O_2_/5% CO_2_. Interneurones in the CA3 *stratum radiatum *and CA3 and CA1 *stratum lacunosum-moleculare *were visualised under IR-DIC optics (Nikon). Membrane currents were recorded by whole-cell voltage-clamp using a Multipatch 700B amplifier and pClamp9 software (Molecular Devices). Patch pipettes had tip resistances of 5–10 MΩ when filled with internal solution: 130 mM KMeSO_4 _or CsMeSO_4_, 4 mM NaCl, 10 mM HEPES, 0.5 mM EGTA, 4 mM Mg-ATP, 0.2 mM Na_2_GTP; pH 7.3, 300 mOsmol/kg. Pressure micro-application of *myo*-inositol (5–15 p.s.i.; Picospritzer, Warner Instruments) was achieved using a glass pipette of tip diameter 50–150 μm positioned adjacent to the target cell body. All test compounds were bath perfused via gravity feed. In some experiments, brain slices were stimulated by: perfusion of glutamate (10 or 100 μM) for 5–30 min; phorbol 12-myristate 13-acetate (PMA) (0.5 or 5 μM) for 30–60 min; D-*myo*-phosphatidylinositol 4,5-bisphosphate-sn-1,2-di-O-octanoyl-glyceryl, 3-O-phospho linked (DiC8) (10 μM in the intracellular solution allowed to dialyse for at least 15 min before recording); 25 mM K^+ ^external solution for 2 min; pre-incubation of slices at 30°C (for >2 h before recording); or depolarisation by current injection in current-clamp mode.

Data analysis of agonist-evoked currents was performed using pClamp9 (Molecular Devices) and Origin7.5 (Original Lab Corp.) software. Concentration-response curves were fitted with the Hill equation: *y *= 1+ (EC_50_/*x*)^*nH*^, where *y *is the membrane current, EC_50 _is the concentration of half-maximal efficacy, *x *is the agonist concentration, and *n*_*H *_is the Hill coefficient. All data are reported as mean ± S.E.M.

### HMIT null-mutant mouse generation

The technical strategy for the KO mouse generation is described in Additional files 1 and 4.

## Competing interests

The authors declare that they have no competing interests.

## Authors' contributions

EDD and EM experimental design, immunocytochemistry, Western blotting, immunoprecipitation and infra-red immunohistochemistry. MHSM electrophysiology studies. EDD and MHSM analysis of results, writing of manuscript, discussion of experimental results. CM, EZ and LLC immunohistochemistry in the rat and human brain. TJP [^3^H]-inositol uptake and [^3^H]-CDP-DAG accumulation experiments. AJS intracellular *myo*-inositol measurements. GD designed the HMIT KO strategy and coordinated mouse generation. CJL, PRM and JNCK concept and interpretation of study, discussion of experimental results, writing of manuscript, manuscript revision. All authors drafted, read and approved the manuscript.

## Supplementary Material

Additional file 1 **Additional methods and additional figure legends**. Details of additional methods and figure legends provided. Click here for file

Additional file 2 **Anti-HMIT antibody validation**. Western blotting of HEK293 cell lysates following immunoprecipitation; HMIT immunocytochemistry in transfected HEK293 cells, HMIT immunoprecipitation and Western blotting in rat and mouse brain tissue. Click here for file

Additional file 3 **Analysis of rat saggital slices stained with anti-HMIT:21 antibody**. Results of infra-red analysis of HMIT localisation in the rat brain. Click here for file

Additional file 4 **Generation of HMIT null-mutant mice**. Description of strategy used to generate HMIT null-mutant mice and mRNA analysis in neurons. Click here for file
