# Supplementary material for: Evaluation of expression and function of the H+/myo-inositol transporter HMIT
Source: BMC Cell Biol. 2009 Jul 16;10:54. doi: 10.1186/1471-2121-10-54 (PMC2717050; doi:10.1186/1471-2121-10-54)
Supplement: Additional file 1 — Additional methods and additional figure legends. Details of additional methods and figure legends provided. [file 1471-2121-10-54-S1.doc]

Additional Methods

**Immunoprecipitation**

HEK293 cells were transfected with pcDNA3.1/V5-His-TOPO-HMIT (HMIT-V5 cDNA) plasmid and lysed 2 days post-transfection in a mild lysis buffer consisting of 50 mM Tris-HCl pH 7.5, 150 mM NaCl, 0.5% (v/v) Triton X-100, 1 mM EDTA, protease and phosphatase inhibitors (Sigma, Protease Inhibitor Cocktail used at 1:200; Phosphatase Inhibitor Cocktail 2 used at 1:100). The formation of the antibody-antigen complex was carried out using 500 μl of cell lysate together with specific antibodies (1-5 μg of antibody per sample) directed against either HMIT (anti-HMIT:21) or the V5 epitope (Serotec); repeated samples were incubated at 4°C on a rotating wheel overnight with 25 μl bed volume of Protein G Sepharose beads (Sigma). The complex was spun briefly and the supernatant carefully removed and discarded. The beads were washed twice with protein lysis buffer followed by one wash with 1x Tris-buffered saline (TBS). Proteins were eluted by boiling the beads for 5 min at 100^o^C in 35 μl of 2x protein sample buffer (4% (w/v) SDS, 20% (v/v) glycerol, 50 mM TRIS pH 6.8, 2% (w/v) bromophenol blue, 2% (v/v) β-mercaptoethanol).

**Western blotting**

Rat/mouse brain or transfected HEK293 cells were lysed with lysis buffer (2% (w/v) SDS, 10% (v/v) glycerol, 25 mM TRIS pH 6.8, 0.01% (w/v) bromophenol blue, 1% (v/v) β-mercaptoethanol). Samples were resolved by SDS polyacrylamide gel electrophoresis (SDS-PAGE) using 4-20% (w/v) gels (Novex). Gels were wet-blotted onto a nitrocellulose (Amersham) or PVDF (Millipore-Immoblion FL) membrane in TG buffer (25 mM Tris pH 8.3, 192 mM glycine; BioRad) including 20% (v/v) methanol at 20 V overnight. Following blotting, the membrane was blocked with Odyssey blocking buffer diluted 1:2 in PBS (Li-Cor) for 1 h to prevent non-specific binding of the primary antibody. The primary antibodies anti-HMIT:21 or anti-V5 were added to the membrane (diluted in blocking buffer) and incubated overnight at 4ºC. The secondary antibody was then added for 1 h at RT after three 10 min washes with PBST (PBS plus 0.05% (v/v) Tween 20). The infrared Odyssey detection system (Li-Cor) required the use of secondary antibodies detected by the infra-red system, which were anti-mouse or anti-rabbit (Li-Cor) (680 or 800), diluted at 1:10,000 in Odyssey blocking buffer containing 1% (v/v) SDS and 0.1% (v/v) Tween 20. The membrane was washed three times with PBST and visualised using the Odyssey imaging system.

**Infra-red immunohistochemistry**

Tissue sections were first incubated for 45 min in Odyssey blocking solution, then incubated with anti-HMIT:21 or the peptide pre-incubated with the antibody overnight at 4°C with gentle shaking. The sections were washed in TBST (TBS plus 0.05% (v/v) Tween 20) four times for 15 min. Sections were then incubated with the secondary anti-rabbit 800 antibody (Li-Cor; 1:10,000) for 1 h at RT. Following this incubation, sections were washed four times in TBST for 30 min per wash, then rinsed in distilled water and mounted on slides without coverslips. The sections were imaged with the Odyssey imaging system using 21 µm resolution, 1 mm offset and medium quality.

**Generation of the HMIT targeting construct**

The HMIT targeting construct was generated in such a manner as to allow the production of either a null allele or the conditional allele upon breeding the primary targeted allele (PTA) to mice expressing either Cre or Flp recombinase respectively.

Using Phusion high fidelity PCR polymerase (Finnzyme) two arms of homology and an exonic region encompassing exon 2 of the HMIT locus were amplified from C57Bl/6J derived genomic DNA. The 3.7 kb 5’ and 3.8 kb 3’ arms of homology, corresponding to intronic regions flanking the second exon, were amplified using the following phosphorylated primer sets: for the 5’ arm, 5’F–CCACAAGCTTGGTGTGGGGG and 5’R–GGGGTCATGAACACAATGGG; and for the 3’ arm, 5’F–GGCTTAAAGGTGGGGCACAGC and 5’R–GGGCACTGACCACTCTTCC. The 744 bp fragment encompassing the second exon was amplified using the phosphorylated primer set 5’F-CCCATTGTGTTCATGACCCC and 5’R-CGAAAGGAAGCTAGGCCTGC. The resulting PCR amplicons were blunt end cloned into a variant of pBluescript II SK (Stratagene) containing a 5’ isolated Lox P element, a phosphoglycerate kinase promoter driven neomycin phosphotransferase gene flanked by Lox P and FRT elements and the MC1 driven diphtheria toxin A gene to generate the targeting construct (see Additional file 4).

**Generation of HMIT null-mutant mice**

Hybrid F1 (ESAR F1 R9 7, Rosa26^(SA-creER)^; Artemis Pharmaceuticals GmbH) embryonic stem (ES) cells were electroporated with 30 μg of linearised targeting vector. Homologous recombination in neomycin resistant ES cells following G418 antibiotic selection was confirmed by Southern blot analysis of BamH I (see Additional file 4 B I) digested genomic DNA hybridised with a probe (see square black box Additional file 4A) external to the 5’ homologous region. The presence of the isolated Lox P element upstream of exon 2 was confirmed by PCR analysis (see Additional file 4 B II) using the primer set 5’F–GGCTTGAGCCAAGCATTAGG and 5’R–ggcacgtaggtgaagcagta (designated a and b in Additional file 4A). A single targeted ES cell clone was injected into BalB C host blastocysts. The resulting male chimeras were bred to the constitutive Cre deletor line C56Bl/6 ROSA26-(SA-CrepA)ARTE (Artemis Pharmaceuticals GmbH) to achieve germline transmission of the null allele. HMIT ^-/+^ mice were then bred for one generation with C57BL/6J mice. Male and female HMIT ^-/+^ mice were then intercrossed and the resulting N2F1 homozygous mice were bred to generate N2F2 homozygote null E15 embryos.

**Taqman HMIT null-mutant mice**

Taqman RT-PCR was performed as described by Di Daniel *et al.* [3]. The following primers and probes were used:

FW 5'-GCTGCCTCCCTGAAACCAAG-3'

REV 5'-AGCGCCACATGAACATAGCC-3'

PROBE 5'-FAM-TGGAGGAAATCGAATCGCTCTTTGACCA-3'-TAMRA

**Additional Figure Legends**

**Additional file 2**

**A Western blotting (WB) of HEK293 cell lysates following immunoprecipitation (IP)**. Cells were either left untransfected (-) or transfected (+) with pcDNA3.1/V5-His-TOPO-HMIT. Cell lysates were incubated with either anti-V5, anti-HMIT:21 or an unrelated polyclonal antibody (-) during IP. Western blotting was performed using anti-HMIT:21 antibody. Membranes were incubated with infra-red secondary anti-rabbit 680 antibody. A strong band was visible at the expected size for HMIT protein, ~80 kDa, in transfected cells, and a weaker band of the same size (representing endogenous HMIT) was observed in untransfected cells. Moreover, higher molecular weight bands representing glycosylated HMIT are visible on the blots.

**B HMIT Immunocytochemistry in transfected HEK293 cells.** Cells were transfected with pcDNA3.1/V5-His-TOPO-HMIT, fixed two days post-transfection and stained with anti-HMIT:21 antibody in combination with an anti-V5 antibody. The secondary antibodies used were Alexa Fluor anti-rabbit 488 and anti-mouse 594. Coverslips were mounted with ProLong Gold with DAPI (blue) to label the cell nuclei. Fluorescence images were acquired using an Olympus BX51 fluorescence microscope and a 60x oil objective. Co-staining with anti-HMIT (green) and anti-V5 (red) antibodies showed that the anti-HMIT:21 antibody specifically recognised overexpressed HMIT-V5 (yellow merged cells). Weak HMIT staining was also observed in untransfected cells, representing endogenous human HMIT protein, which was detected by the antibody as it was designed to recognise both human and rat HMIT.

**C HMIT immunoprecipitation and Western blotting in rat and mouse brain tissue**. Lysates were incubated with either anti-HMIT:21 antibody (+) or an unrelated polyclonal antibody (-). The membrane was incubated with anti-HMIT:21 antibody followed by the secondary infra-red anti-rabbit 680 and imaged using the infra-red Odyssey system. HMIT protein was expressed in rat and mouse brain, as visualised by the band at ~80 kDa. The use of an unrelated polyclonal antibody in the IP experiment as a negative control showed that the anti-HMIT:21 antibody is specific.

**Additional file 3**

**Analysis of rat saggital slices stained with anti-HMIT:21 antibody**.

Slices (40 μm thick) were stained with anti-HMIT:21 antibody followed by infra-red secondary antibody anti-rabbit 800. Slices were mounted on glass slides and imaged using the Odyssey infra-red detection system. Image **A** shows that HMIT is present in most brain regions, with a high level of expression in the hippocampus. Image **B** is an enlargement of the hippocampus, showing high expression in the CA2, CA3 and dentate gyrus (DG) regions.

**Additional file 4**

**Generation of HMIT null-mutant mice.**

**A** Schematic representation of the first 3 exons of the HMIT wild-type locus, HMIT targeting construct, primary targeted allele (HMIT^PTA/+^) and the HMIT null allele

(HMIT ^-/+^). The 5’ external probe used for Southern blot analysis of BamH I (**B** **I**) digested genomic DNA is represent by the square black box.

**B** Confirmation of targeting at the HMIT locus. **I**) Southern blot analysis of BamH I digested genomic DNA hybridised with a 5’ external probe (**A**). In addition to the wild-type fragment of approximately 21 kb, a smaller, approximately 7 kb fragment was observed for the targeted allele in targeted ES cell derived genomic DNA. **II**) PCR amplification across the 5’ isolated Lox P element. Amplification using primers a and b (**A**) on wild-type C57Bl/6J DNA generated a 181 bp amplicon, while amplification of DNA from the HMIT^PTA/+^ ES cell resulted in the generation of both the 181 bp and a larger 294 bp mutant amplicon, due to the insertion of the isolated Lox P element.

**C** Taqman RT-PCR of E15 neurones from WT (HMIT ^(+/+)^) and KO (HMIT ^(-/-)^) mice (n = 1 cell preparation, 2 wells/genotype).
