# Supplementary figures and images for: Evaluation of expression and function of the H+/myo-inositol transporter HMIT
Source: BMC Cell Biol. 2009 Jul 16;10:54. doi: 10.1186/1471-2121-10-54 (PMC2717050; doi:10.1186/1471-2121-10-54)

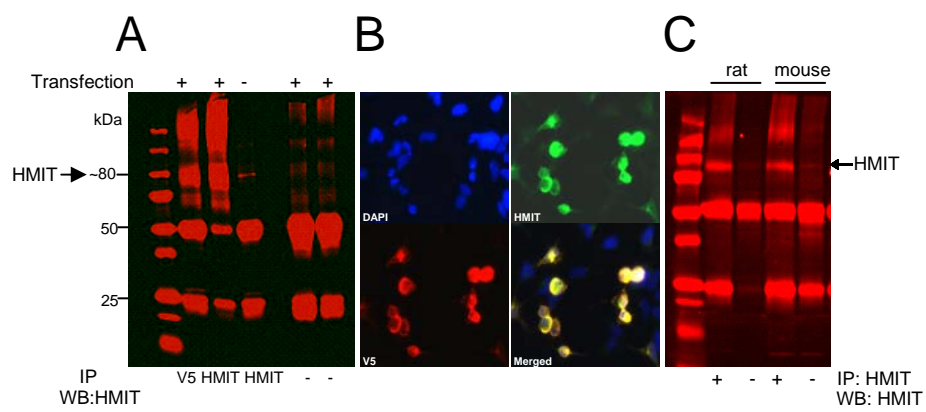

Supplement: Additional file 2 — Anti-HMIT antibody validation. Western blotting of HEK293 cell lysates following immunoprecipitation; HMIT immunocytochemistry in transfected HEK293 cells, HMIT immunoprecipitation and Western blotting in rat and mouse brain tissue. [file 1471-2121-10-54-S2.pdf]

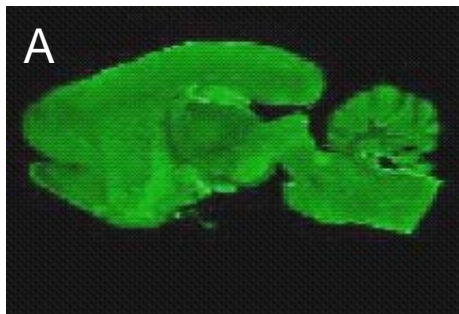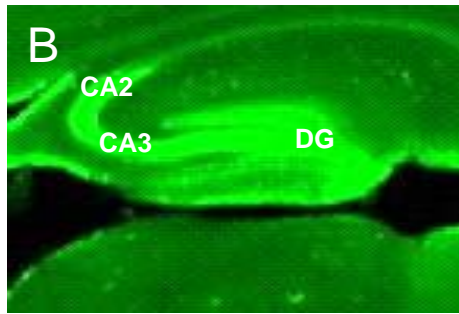

Supplement: Additional file 3 — Analysis of rat saggital slices stained with anti-HMIT:21 antibody. Results of infra-red analysis of HMIT localisation in the rat brain. [file 1471-2121-10-54-S3.pdf]

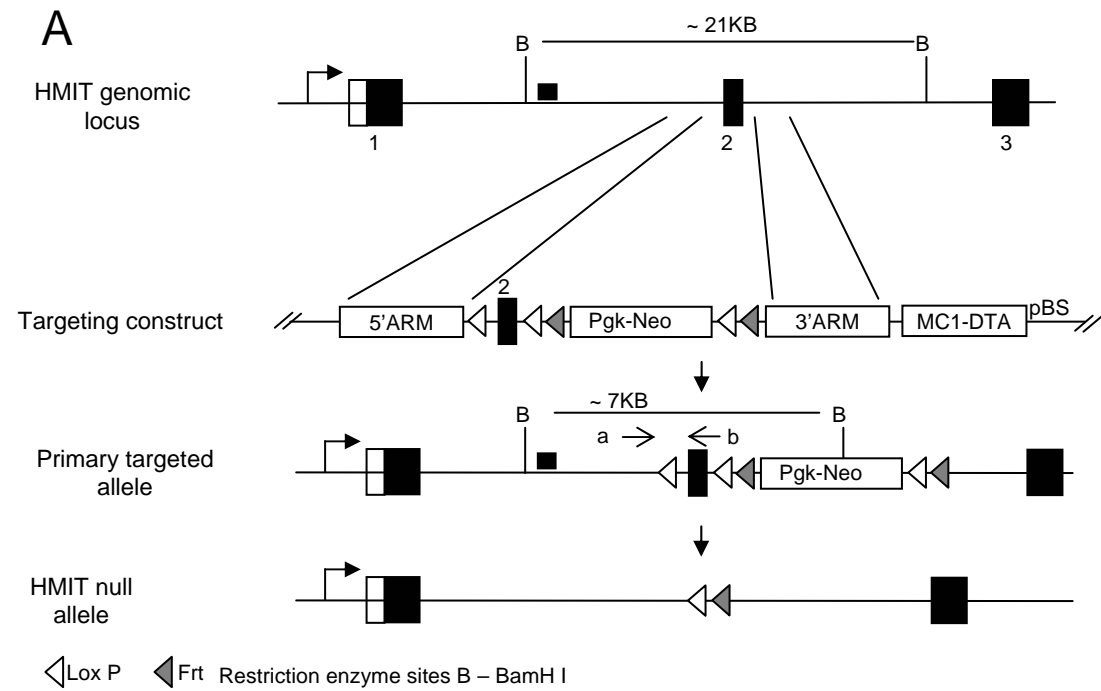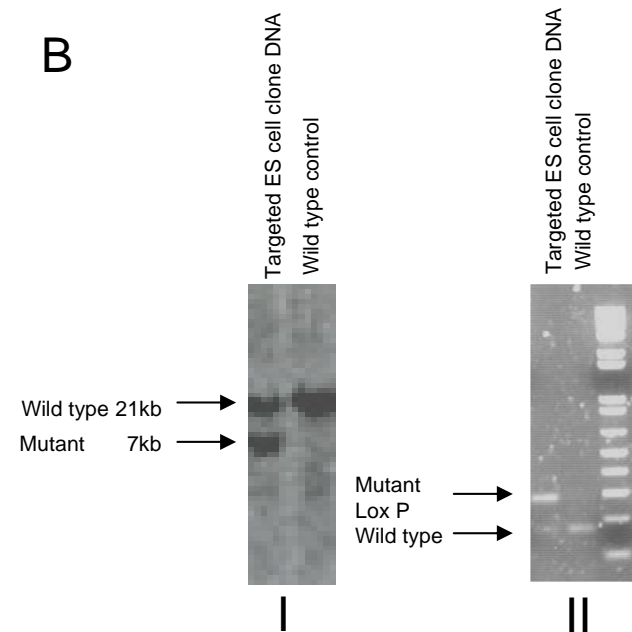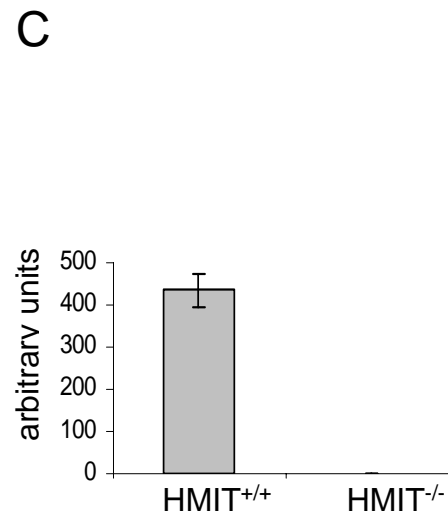

Supplement: Additional file 4 — Generation of HMIT null-mutant mice. Description of strategy used to generate HMIT null-mutant mice and mRNA analysis in neurons. [file 1471-2121-10-54-S4.pdf]
